# Early gut microbiota signature of aGvHD in children given allogeneic hematopoietic cell transplantation for hematological disorders

**DOI:** 10.1186/s12920-019-0494-7

**Published:** 2019-03-07

**Authors:** Elena Biagi, Daniele Zama, Simone Rampelli, Silvia Turroni, Patrizia Brigidi, Clarissa Consolandi, Marco Severgnini, Eleonora Picotti, Pietro Gasperini, Pietro Merli, Nunzia Decembrino, Marco Zecca, Simone Cesaro, Maura Faraci, Arcangelo Prete, Franco Locatelli, Andrea Pession, Marco Candela, Riccardo Masetti

**Affiliations:** 1 Department of Pharmacy and Biotechnology, University of Bologna, Via Belmeloro 6, 40126 Bologna, Italy; 2 Pediatric Oncology and Hematology Unit “Lalla Seràgnoli”, Department of Pediatrics, University of Bologna, Sant’Orsola Malpighi Hospital, Via Massarenti 9, 40138 Bologna, Italy; 3 Institute of Biomedical Technologies, Italian National Research Council, Via Fratelli Cervi 93, 20090 Segrate, Milan Italy; 4 Department of Pediatric Hematology/Oncology, IRCCS Ospedale Pediatrico Bambino Gesù, Rome, Italy; 5 Division of Pediatric Hematology/Oncology, Fondazione IRCCS Policlinico San Matteo, Pavia, Italy; 6 Azienda Ospedaliera Universitaria Integrata di Verona, Verona, Italy; 7 Hematopoietic stem cell Unit, Department of Hematology-Oncology, IRCCS-Istituto Gaslini, Genoa, Italy

**Keywords:** Gut microbiota, Hematopoietic stem cell transplantation, Acute graft-versus-host disease, Alloreactivity, 16S rRNA gene sequencing, Pediatric patients

## Abstract

**Background:**

The onset of acute Graft-versus-Host Disease (aGvHD) has been correlated with the gut microbiota (GM) composition, but experimental observations are still few, mainly involving cohorts of adult patients. In the current scenario where fecal microbiota transplantation has been used as a pioneer therapeutic approach to treat steroid-refractory aGvHD, there is an urgent need to expand existing observational studies of the GM dynamics in Hematopoietic Stem Cell Transplantation (HSCT). Aim of the present study is to explore the GM trajectory in 36 pediatric HSCT recipients in relation to aGvHD onset.

**Methods:**

Thirty-six pediatric patients, from four transplantation centers, undergoing HSCT were enrolled in the study. Stools were collected at three time points: before HSCT, at time of engraftment and > 30 days following HSCT. Changes in the GM phylogenetic structure were studied by 16S rRNA gene Illumina sequencing and phylogenetic assignation.

**Results:**

Children developing gut aGvHD had a dysbiotic GM layout before HSCT occurred. This putative aGvHD-predisposing ecosystem state was characterized by (i) reduced diversity, (ii) lower *Blautia* content*,* (iii) increase in *Fusobacterium* abundance. At time of engraftment, the GM structure underwent a deep rearrangement in all patients but, regardless of the occurrence of aGvHD and its treatment, it reacquired a eubiotic configuration from day 30.

**Conclusions:**

We found a specific GM signature before HSCT predictive of subsequent gut aGvHD occurrence. Our data may open the way to a GM-based stratification of the risk of developing aGvHD in children undergoing HSCT, potentially useful also to identify patients benefiting from prophylactic fecal transplantation.

**Electronic supplementary material:**

The online version of this article (10.1186/s12920-019-0494-7) contains supplementary material, which is available to authorized users.

## Background

Allogeneic Hematopoietic Stem Cell Transplantation (HSCT) is a curative option for many patients with high-risk hematopoietic malignancies and hematological disorders. The success of the procedure can be hampered by a process in which donor-derived T cells recognize host healthy tissue as *non-self*, causing an immune-mediated complication known as acute Graft-versus-Host Disease (aGvHD), instrumental in determining morbidity and mortality of the patients.

A relationship between aGvHD and the gut microbiota (GM) has long been hypothesized, [1–3] because of the ever-increasing evidence of the involvement of the GM in the regulation of the human immune system functionality [4, 5]. Few studies, mostly performed in small cohorts, have documented that aGvHD is associated with detectable shifts in the composition of the GM, in both murine and human (adult and child) HSCT recipients [6–8]. This finding supports the hypothesis that GM-derived metabolites (e.g. short-chain fatty acids (SCFAs)) and/or signaling molecules (e.g. microbe-associated molecular patterns (MAMPs)) can influence the onset of aGvHD. Indeed, it has been demonstrated in a mouse model that bacteria-originated butyrate improves the junctional integrity of intestinal epithelial cells, decreases apoptosis and mitigates aGvHD severity [9]. Some of the studies characterizing the GM dynamics in HSCT showed that a specific gut microbial population, the so-called “anti-inflammatory Clostridia”, i.e. members of the families Clostridiaceae, Lachnospiraceae, Ruminococcaceae and Eubacteriaceae, might be involved in the mechanism by which gut microbes exert a counteracting effect on aGvHD onset and progression [8–10]. A partial confirmation of this observation has been provided by a study on a larger cohort of adults, in which the relative abundance of the Lachnospiraceae genus *Blautia* was correlated with reduced mortality from aGvHD [11].

The understanding of the relationship existing between GM and development of aGvHD is still far from complete, mostly because of the cumbersome nature of studies on diseased human subjects. In addition to the difficulty of enrolling patients for observational trials, studies on the GM of HSCT recipients can be biased by many confounding variables, i.e. antibiotic treatment, proton-pomp inhibitors, chemotherapy protocols, underlying malignancy, and hospitalization itself. Still, the need to thoroughly explore this relationship is more compelling than ever, last considering that transplantation of fecal material has been used as a pioneer therapeutic strategy to approach treatment-refractory aGvHD [12]. The aim of the present work is to expand the pilot study on the GM dynamics previously performed in 10 pediatric HSCT recipients in relation to the development of aGvHD [7]. In particular, four transplantation centers across Italy enrolled a total of 36 individuals, with a longitudinal approach to fecal sampling. The analysis, aside from attempting to detect microbial signatures that might be related to the aGvHD onset and/or progression, was aimed at elucidating the extent of the changes occurring in the GM composition in relation to aGvHD severity.

## Methods

### Patients

**Fig. 1:**
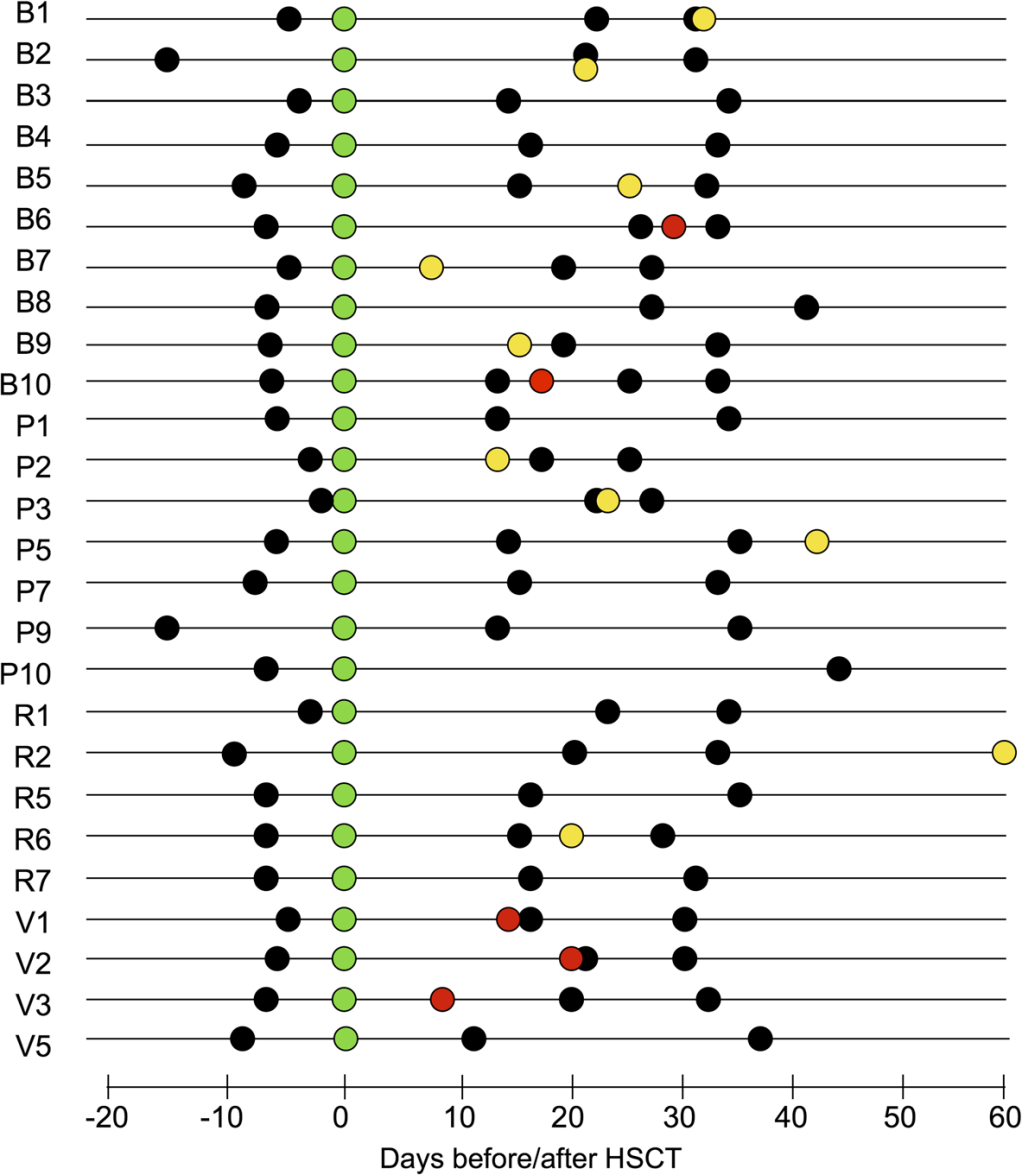
Schematic overview of the sampling time for each enrolled subject. Subjects are indicated with a progressive number preceded by a letter associated with the enrolling center (B, Bologna; P, Pavia; R, Rome; V, Verona). HSCT (green dots), skin grade I-III aGvHD diagnosis (yellow dots), gastrointestinal grade II-IV aGvHD diagnosis (red dots), and fecal sample collection (black dots) are plotted on timelines with distance from HSCT expressed in days

Thirty-six patients (20 male and 16 female) given allo-HSCT in four pediatric centers in Italy (Bologna, Pavia, Rome and Verona) between 2012 and 2016, were enrolled in a stool collection protocol approved by the University of Bologna Ethics Committee (ref. number 19/2013/U/Tess). Written informed consent was obtained, in accordance with the Declaration of Helsinki, from each enrolled patient or parent/legal guardian. Inclusion criterion was the availability of a pre-HSCT fecal sample and of at least two samples collected after HSCT. For one subject, the material collected at time of engraftment was insufficient to extract good-quality bacterial DNA; therefore, only one post-HSCT sample was included (Fig. 1). Patients with either malignant (63% of cases) or non-malignant diseases were included; in particular, eight of 23 patients with malignant disease were classified as having high-risk disease. All transplantation procedures took place in HEPA-filtered single rooms where standard precautionary and hygienic measures were applied. Antibiotic gut decontamination during the conditioning regimen was not routinely performed. Only five out of 36 patients received intravenous levofloxacin [13] from the beginning of the conditioning regimen to the complete neutrophil recovery. All patients observed complete fasting beginning from the onset of oral mucositis or diarrhea with administration of parenteral nutrition until the day of engraftment. As for conditioning regimen, 32 patients received a myeloablative busulfan (or treosulfan)-based conditioning regimen, three patients received total body irradiation-based conditioning regimen and one patient received a non-myeloablative preparative regimen with cyclophosphamide/fludarabine. Antiviral and antifungal prophylaxis was administered from day − 1 and + 2 respectively, as recommended [14, 15]. Antibiotic empiric therapy, with piperacillin/tazobactam or ceftazidime was given in case of neutropenic fever in 29/36 cases. Seven cases received antibiotic therapy at the beginning of the neutropenic phase independently from the onset of fever, and four cases received a cephazolin/piperacillin tazobactam-based anti-streptococcal prophylaxis before the engraftment phase. Two patients developed sepsis, one sustained by *Escherichia coli* before graft infusion and the other by *Klebsiella pneumoniae* after cells infusion and before the neutrophil engraftment. Concerning the types of allograft administered, 29/36 patients received unmanipulated bone marrow cells, while 6/36 patients received T-cell depleted peripheral blood stem cells and 1/36 cord blood cells. Neutrophil and platelet engraftment were defined as occurring on the first of the three consecutive days on which the neutrophil count was > 0.5 × 10^9^/L and platelets were > 20 × 10^9^/L, respectively. Only one patient experienced primary graft failure.During the procedure, 19 of 36 patients developed aGvHD; six had gut involvement. All patients with skin and gastrointestinal aGvHD (grade I-III according to the Seattle criteria) [16] were treated with steroids, while one patient with skin grade III aGvHD also received extracorporeal photopheresis. One patient with severe gastrointestinal grade IV aGvHD was treated with steroids, extracorporeal photopheresis and infliximab.

### Sample collection and bacterial DNA isolation

Stool samples were collected using sterile stool collection tubes, stored at minimum − 20 °C and shipped in dry ice to the laboratory where microbiological analyses were performed. Total bacterial DNA was extracted from 250 mg of each sample following a previously described protocol [17].

### 16S rRNA gene amplification and sequencing

The V3-V4 region of the 16S rRNA gene was PCR amplified, using S-D-Bact-0341-b-S-17/S-D-Bact-0785-a-A-21 primers carrying Illumina overhang adapter sequences, [18] at a final concentration of 200 nmol/L. Twenty-five ng of microbial DNA were used as a template in a final reaction volume of 50 μL. KAPA HiFi HotStart ReadyMix (KAPA Biosystems, Resnova, Italy) was used for the amplification. The thermal cycle was set as follows: 3 min at 95 °C, 25 cycles of 30 s at 95 °C, 30 s at 55 °C, and 30 s at 72 °C, and a final 5-min step at 72 °C. Amplicons were purified with a magnetic bead-based clean-up system (Agencourt AMPure XP; Beckman Coulter, Brea, CA). Indexed libraries were prepared by limited-cycle PCR using Nextera technology, followed by a second AMPure XP magnetic beads clean-up step. Final libraries were prepared by equimolar (4 nmol/L) pooling, denaturation and dilution to 6 pmol/L before loading onto the HiSeq flow cell. A 2 × 250 bp paired-end protocol was used, according to manufacturer’s instructions (Illumina, San Diego, CA).

### Bioinformatics and statistics

Raw sequences were processed using a pipeline combining PANDAseq and QIIME [19, 20]. Sequencing reads were deposited on European Nucleotide Archive (ENA) (http://www.ebi.ac.uk/ena/data/view/PRJEB23820). High-quality reads, as selected using the default values in QIIME, were binned into operational taxonomic units (OTUs) according to the taxonomic threshold of 97% using UCLUST, [21] through an open-reference strategy. For statistical purposes, the OTU tables from subjects included in the study of Biagi et al. [7] were included in the analyses, and taxonomy for all samples was assigned using the RDP classifier against Greengenes database (May 2013 release). Chimeras and singleton OTUs were discarded. Statistics was performed using R software (https://www.r-project.org/) and the libraries vegan, made4, Random Forest, and rfPermute. Since samples from Biagi et al. [7] were sequenced with a different technique (Roche 454 pyrosequencing vs. Illumina) and using a smaller region of the 16S rRNA gene for assignment (V4 only vs. V3-V4), analyses were performed using genus-level relative abundances for Principal Coordinates Analysis (PCoA) based on Bray-Curtis distances. Datasets were filtered for genera present at relative abundance > 0.1% in at least one sample. Data separation in the PCoA was tested using a permutation test with pseudo F ratios (function adonis in the vegan package). Bacterial groups with the largest contribution to the ordination space were found by using the function envfit of the R package vegan on the genus relative abundances. The impact of different variables on the microbiota structure was explored by using the Random Forest machine learning algorithm [22]. Briefly, Random Forests is a powerful classifier that identifies the best subset of features (here, relative abundances at genus level) to discriminate among categories (age groups, studies, hospitals, types of hematological disease). Wilcoxon test was used to assess significant differences between two groups of samples; adaptations for paired samples were used when necessary. Kruskal-Wallis test was used for multiple comparisons, followed by Tukey post-hoc test when appropriate. All *P* values were corrected for multiple comparisons using the Benjamini-Hochberg method (the number of hypotheses tested for each comparison ranged between 40 and 64). A false discovery rate (FDR) of 5% was used. Finally, sequences assigned to a selected group of taxa, namely *Enterococcus, Citrobacter, Erwinia, Fusobacterium, Blautia,* and *Bacteroides*, were subjected to additional analysis using the tool Oligotyping [23]. Following the initial entropy analysis, oligotyping was performed using version 2.1 of the Oligotyping pipeline with a total of 5 positions with high entropy, chosen to compute the oligotypes (−C option). To minimize the impact of sequencing errors, we imposed that each oligotype must have at least 10 reads belonging to the most abundant unique sequence. Species-level assignment of the obtained oligotypes was carried out using BLAST, with the representative sequences of each oligotype as a query against the whole 16S rRNA gene sequence NCBI database.

## Results

The 16S rRNA gene-based GM phylogenetic composition was obtained by Illumina sequencing of a total of 78 fecal samples, taken from 26 subjects, enrolled in four Italian hospitals. A mean of 11,325 ± 4480 high quality sequences per sample was obtained. Fifty-three samples from 10 subjects enrolled at the Sant’Orsola-Malpighi hospital of Bologna, sequenced in our previous study on the same topic, [7] were also included in the analysis, obtaining a final cohort of 131 samples from 36 subjects. Clinical and transplant characteristics are summarized in Table 1. Samples were grouped as “pre” (i.e. samples taken before HSCT), “engraftment” (i.e. the first sample taken after HSCT for all subjects, corresponding approximately to the time of hematopoietic engraftment, ranging from 12 to 28 days after HSCT), and “post” (all other samples taken after HSCT) (Fig. 1).

**Table 1:** Anagraphical and clinical information characteristics of the enrolled patients

Characteristics	N	(%)	aGvHD	No aGvHD	P
Age (years)					ns
Mean	8.4		8.2	9.0	
Range	1–21		2–19	1–21	
Female Sex	12	33.3	6	6	
Disease					ns
ALL	14	38.9	7	7	
AML	9	25.0	4	5	
Thalassemia Major	5	13.9	2	3	
SAA	3	8.3	2	1	
BDA	3	8.3	2	1	
CDA	1	2.8	1	0	
ALPS	1	2.8	1	0	
Risk^a^					ns
High Risk	8	22.2	5	3	
Standard Risk	28	77.8	14	14	
Graft Source					ns
Unmodified BM	29	80.6	16	13	
T-cell Depleted PBSC	6	16.7	2	4	
Cord Blood	1	2.8	1	0	
Matching HLA					ns
Matched	26	72.2	14	12	
Mismatched	10	27.8	5	5	
Conditioning Regimen^b^					ns
Myeloablative	35	88.9	18	16	
Nonmyeloablative	1	11.1	1	0	
GvHD Prophylaxis					ns
αβ T-cell and B-cell depletion	6	16.7	3	3	
Calcineurin Inhibitor alone	7	19.4	4	3	
CI + ATG +/− MTX	21	58.3	11	10	
CI + ATG + Prednisone	1	2.8	1	0	
CI + Cyclophosphamide post	1	2.8	0	1	
GvHD	19	52.8			
Mean Day of Onset (days from transplantation)	+ 21				
I	5	26.3			
II	9	47.4			
III	3	15.8			
IV	2	10.5			
Gut Involvement	6	31.6			

Abbreviations: *ALL* Acute Lymphoblastic Leukemia, *AML* Acute Myeloid Leukemia, *SAA* Severe Aplastic Anemia, *BDA* Blackfan-Diamond Anemia, *CDA* Congenital Dyserythropoietic Anemia, *ALPS* Autoimmune Lymphoproliferative Syndrome, *BM* Bone Marrow, *PBSC* Peripheral Blood Stem Cells, *GvHD* Graft-versus-Host Disease, *ATG* Anti-Thymocyte Globulin, *MTX* Methotrexate, *ns* not significant

^a^Risk staging: *high risk*: >1st complete remission (CR) or refractory disease; *standard risk*: 1st CR or non-malignant disease

^b^As defined by Bacigalupo et al. [50]

### Exploration of confounding variables

Principal Coordinates Analysis (PCoA) based on Bray-Curtis distances between genus-level microbial profiles showed no separation between the samples sequenced in the present work and those from our previous study [7] (adonis test, *P* > 0.05) (Additional file 1: Figure S1). However, according to the Random Forest analysis, the relative abundance of some bacterial genera was discriminatory between studies (*P* < 0.01, error rate 4.6%), namely *Streptococcus* (average 0.7 and 6.2% in the 2015 study and in the present study, respectively), *Erwinia* (0 and 6.8%), *[Eubacterium]* (6.1 and 0.8%), and *[Ruminococcus]* (6.3 and 3.8%). Mentioned taxa were not excluded from subsequent analysis, but caution was taken in associating them with other variables, because of their possible relation to the different sequencing techniques used in the two studies (Roche 454 pyrosequencing of the 16S rRNA gene V4 hypervariable region in the 2015 study, and Illumina sequencing of the 16S rRNA gene V3-V4 hypervariable regions in the present study).

According to the Random Forest analysis, the genus-level GM profile did not significantly discriminate between age groups, i.e. “baby” (< 2 years), “pediatric” (2–12 years), and “teen” (> 12 years) (error rate, 37%). PCoA analysis confirmed the overlapping between samples from pediatric and teen groups, in all considered group of samples (pre, engraftment and post). However, a separation of the samples taken from the subjects in the “baby” group was revealed in pre and post sample groups (adonis test, *P* = 0.002 and *P* = 0.006, respectively) (Additional file 1: Figure S2). The latter results highlight the loss, and the subsequent recovery, of age-specific GM features following HSCT.

Random Forest analysis also confirmed that the hospital where patients were recruited was an important variable impacting on the genus-level GM profile, with the Bologna hospital being the more easily discriminated (error rate, 16%). PCoA analysis confirmed the impact of the hospitalization center on all considered group of samples (pre, engraftment and post) (adonis test, *P* = 0.003, *P* = 0.001 and *P* = 0.001, respectively) (Additional file 1: Figure S3). The relative abundances of *Enterococcus, Citrobacter, Erwinia*, as well as of unclassified members of Aerococcaceae and Clostridiales, were discriminatory for samples taken in the different hospitals (*P* < 0.01). As mentioned above, these taxa were not excluded from subsequent analysis, but caution was taken in associating them with other variables because of their possible link with the different enrollment centers. Indeed, a detailed analysis (Additional file 1: Figure S4) showed that OTUs assigned to *Enterococcus* were more relatively abundant in the GM of subjects enrolled in the Pavia, Verona and Rome hospitals, with respect to the Bologna hospital, especially in samples taken approximately at engraftment. All oligotypes obtained from the *Enterococcus*-assigned OTUs showed equal identity score to different *Enterococcus* species (i.e. *Enterococcus hirae, Enterococcus faecium, Enterococcus ratti, Enterococcus villorum*), as expected based on the very high similarity among the 16S rRNA gene sequences of the species belonging to this genus [24]. Samples from Verona and Rome hospitals also showed the highest relative abundances of *Citrobacter* (in particular in samples taken before transplantation and after engraftment) and *Erwinia* OTUs. Both *Citrobacter* and *Erwinia* are members of the Enterobacteriaceae family. *Erwinia* is not commonly reported as a component of the human GM. It is known that 16S rRNA gene sequencing, while effective for family-level phylogenetic reconstruction, may underperform for genus-level phylogenetic analysis in the Enterobacteriaceae, [25] suggesting that the detection of high percentages of OTUs assigned to these genera in some samples may indicate hyper-proliferation of an array of common enteric pathogens. Therefore, oligotyping analysis was carried out on sequences assigned to these genera, and representative sequences were re-assigned using NCBI 16S rRNA gene sequence database. Oligotypes obtained from the *Citrobacter*-assigned OTUs showed the highest identity score to one of three commonly reported nosocomial pathogens, i.e. *Citrobacter murliniae, Klebsiella pneumoniae,* and *Enterobacter cloacae* [26–29]. None of these species was associated with a specific hospital or one of the three considered time points (pre, engraftment and post). On the contrary, the majority of oligotypes obtained from the *Erwinia*-assigned OTUs showed the highest identity score to *E. cloacae* (72% of the sequences), and a smaller fraction to *K. pneumoniae*. In particular, in all samples where a high percentage of *Erwinia*-assigned OTUs was detected (> 10% of the whole microbiota, all from Verona and Rome hospitals), the oligotypes assigned to *E. cloacae* were dominant (93% of the sequences on average).

Finally, a possible effect of the underlying disease on the GM composition prior to HSCT was investigated. According to our findings, Random Forest analysis did not discriminate among subjects affected by different hematological diseases on the basis of the pre-HSCT GM composition (error rate, 66.7%).

### Analysis of microbiota variability before HSCT in relation to aGvHD development

**Fig. 2:**
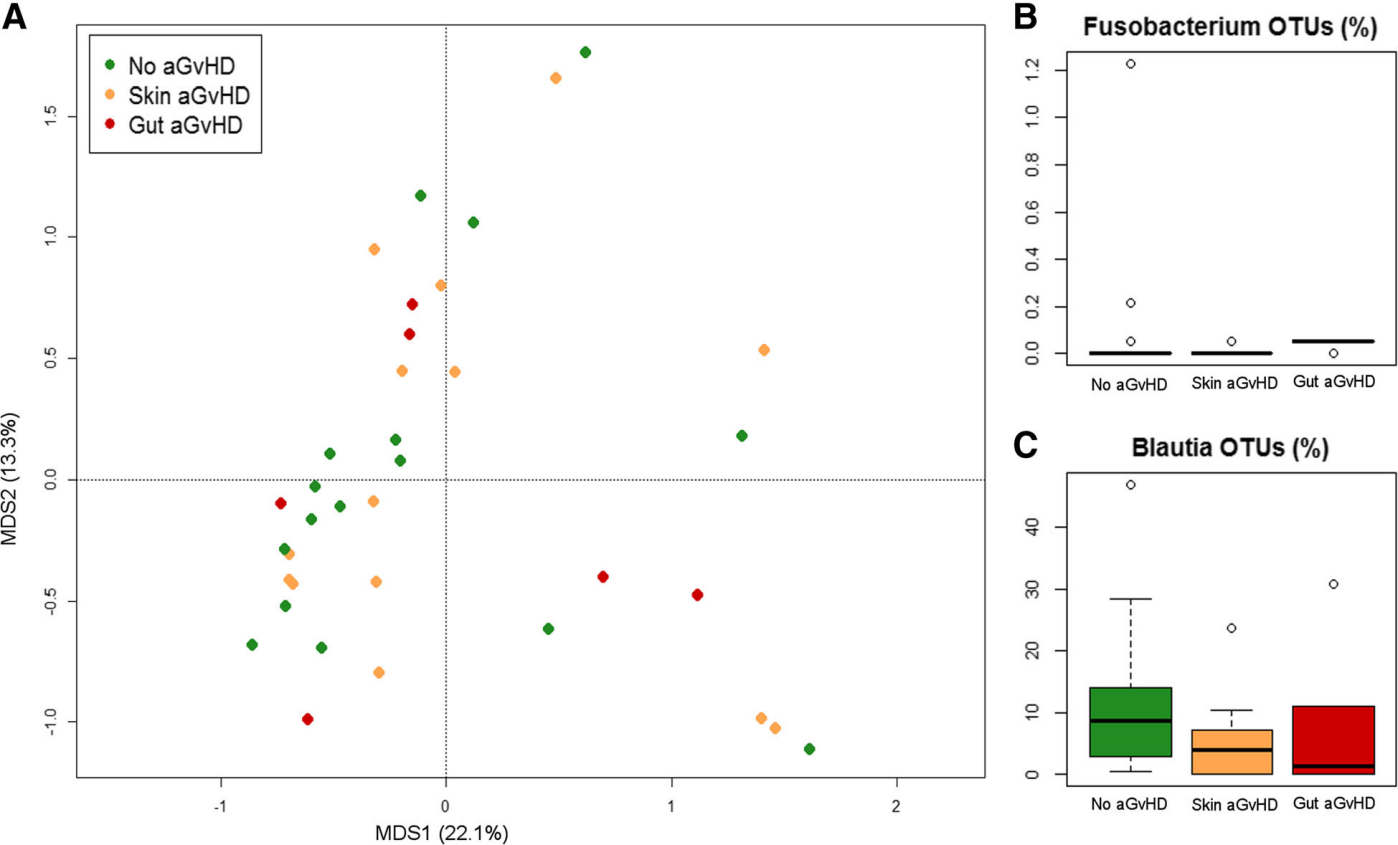
Gut microbiota variability before HSCT, in relation to aGvHD development. **a** PCoA based on Bray-Curtis distances of genus-level relative abundance profiles of samples collected before HSCT from patients who did not develop aGvHD (green), who developed aGvHD (I-II grade) only involving the skin (gold), and who developed gastrointestinal aGvHD (II-IV grade) (dark red). Samples are identified by filled circles. First and second principal components (MDS1 and MDS2) are plotted, accounting for 22.1 and 13.3% of variance in the dataset, respectively. Box and whiskers distribution of the relative abundance (%) of OTUs assigned to the genera *Fusobacterium* (**b**) and *Blautia* (**c**) in samples collected before HSCT from subjects who did not develop aGvHD (green), who developed aGvHD (I-II grade) only involving the skin (gold), and who developed gastrointestinal aGvHD (II-IV grade) (dark red)

PCoA analysis based on genus-level relative abundances showed no separation between samples taken before HSCT from subjects who subsequently either did or did not develop aGvHD (adonis test, *P* > 0.05) (Fig. 2a). However, pre-HSCT samples from subjects who did not develop aGvHD tended to show higher relative abundance of OTUs assigned to the genus *Blautia* (mostly assigned to the human gut species *Blautia wexlerae*, according to our oligotyping analysis) compared to subjects who developed any grade of aGvHD (median 8.7% in non-aGvHD, 4.0% in skin grade I-II aGvHD, and 1.4% in gut grade II-IV aGvHD; Wilcoxon test, *P* = 0.046) (Fig. 2c). Conversely, subjects who developed greater severity aGvHD with gastrointestinal involvement showed significantly higher relative abundance of *Fusobacterium*-assigned OTUs with respect to other samples (median 0% in non-aGvHD and skin grade I-II aGvHD, 0.05% in gut grade II-IV aGvHD; Kruskal-Wallis test, *P* = 0.01) (Fig. 2b), as well as higher prevalence, i.e. percentage of samples in which OTUs assigned to *Fusobacterium* were found above the detection limit (19% in non-aGvHD, 14% in skin grade I-II aGvHD, and 83% in gut grade II-IV aGvHD). Based on oligotyping analysis and subsequent matching against the NCBI 16S rRNA gene database, all OTUs assigned to this genus belonged to the species *Fusobacterium nucleatum*. Both *Fusobacterium* and *Blautia* positive and negative association with aGvHD development and localization were confirmed by Random Forest analysis (*P* = 0.006 and *P* = 0.046, respectively). The same analysis allowed identifying a few highly subdominant genera that were detected, even at very low relative abundance, exclusively in pre-HSCT samples of subjects who did not develop aGvHD (i.e. *Leptotrichia* and unclassified members of Barnesiellaceae, average relative abundance 0.02% in non-aGvHD only, *P* = 0.035 and *P* = 0.042, respectively).

The pre-HSCT GM biodiversity was found lower in both gut grade II-IV aGvHD (Simpson index, mean ± SD = 0.70 ± 0.28) and skin grade I-II aGvHD (0.75 ± 0.16) groups of subjects, with respect to subjects who did not develop aGvHD (0.79 ± 0.16) (Additional file 1: Figure S5), although this difference was not statistically significant. As for the inter-individual variability, the diversity between pre-HSCT samples, as approximated by calculation of Bray-Curtis distances, was higher in subjects developing gut aGvHD (Bray-Curtis distances, mean ± SD = 0.86 ± 0.15) than in both non-aGvHD subjects (0.72 ± 0.15, Wilcoxon test *P* = 0.001) and subjects developing lower-severity skin-only aGvHD (0.77 ± 0.15, *P* = 0.02) (Additional file 1: Figure S6).

### Analysis of microbiota variability at engraftment in relation to aGvHD development

**Fig. 3:**
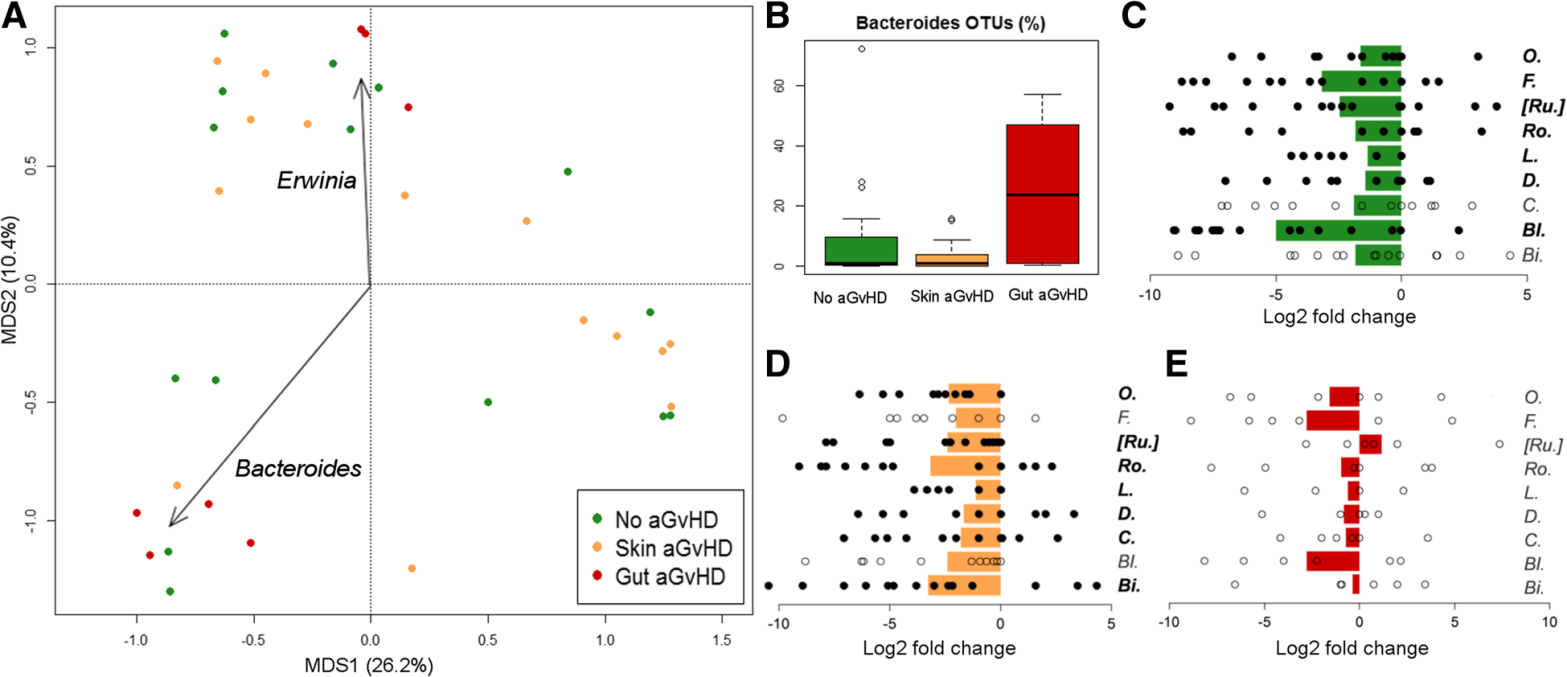
Gut microbiota variability at engraftment, in relation to aGvHD development. **a** PCoA based on Bray-Curtis distances of genus-level relative abundance profiles of samples collected at the engraftment from patients who did not develop aGvHD (green), who developed aGvHD (I-II grade) only involving the skin (gold), and who developed gastrointestinal aGvHD (II-IV grade) (dark red). Samples are identified by filled circles. First and second principal components (MDS1 and MDS2) are plotted, accounting for 26.2 and 10.4% of variance in the dataset, respectively. The biplot of the average bacterial coordinates weighted by the corresponding bacterial relative abundance per sample was superimposed on the PCoA plot for two of the bacterial genera contributing to the ordination space (arrows). **b** Box and whiskers distribution of the relative abundance (%) of OTUs assigned to the genus *Bacteroides* in samples collected before HSCT from subjects who did not develop aGvHD (green), who developed aGvHD (I-II grade) only involving the skin (gold), and who developed gastrointestinal aGvHD (II-IV grade) (dark red). Log2 fold changes of the main discriminant genera between pre-HSCT and engraftment samples in subjects who did not develop aGvHD (**c**), who developed aGvHD (I-II grade) only involving the skin (**d**), and who developed gastrointestinal aGvHD (II-IV grade) (**e**). Genera for which P ≤ 0.05 was obtained for at least one of the three groups of subjects are shown (please see Additional file 2: Table S1) (*O., Oscillospira; F., Faecalibacterium; [Ru.], [Ruminococcus]; Ro., Roseburia; L., Lachnospira; D., Dorea; C., Coprococcus; Bl., Blautia; Bi., Bifidobacterium*). Average values of log2 ratios are reported as bar plot, whereas dots identify the single values obtained for each subject included in each group. Filled black dots and bacterial names in bold are used when the abundance of the OTUs assigned to that genus was significantly different between pre-HSCT and engraftment samples (paired Wilcoxon test, *P* < 0.05), whereas empty circles are used when the difference was not significant

PCoA analysis based on genus-level relative abundances, in samples collected at engraftment, showed that samples from subjects who developed grade II-IV aGvHD with gastrointestinal involvement tended to separate from all other samples (adonis test, *P* = 0.049) (Fig. 3a). Two gut aGvHD samples from two children enrolled at the Verona hospital (subject code, V2 and V3, Fig. 1) were characterized by > 80% of the OTUs assigned to the genus *Erwinia*, and re-assigned by oligotyping analysis and NCBI 16S rRNA gene database to the species *E. cloacae*, and they were plotted distant to the others. However, since OTUs assigned to the genus *Erwinia* were among those listed as possibly influenced by both the sequencing technique and the hospital in which patients were enrolled (Additional File 1: Figure S4), this data might be biased and was not further explored. Engraftment samples of patients with gut grade II-IV aGvHD tended to show higher relative abundance of *Bacteroides* (median 0.7% in non-aGvHD, 0.8% in skin grade I-II aGvHD, and 23.7% in gut grade II-IV aGvHD; Kruskal-Wallis test, *P* = 0.050) (Fig. 3b). According to our oligotyping analysis, the *Bacteroides* species composition showed high variability among individuals, with different proportion of the most common human gut *Bacteroides* species, such as *Bacteroides ovatus, Bacteroides vulgatus, Bacteroides uniformis,* and *Bacteroides dorei*. We could not observe any association between the detection of a specific *Bacteroides* species and the onset of gut grade II-IV aGvHD.

The comparison of pre-HSCT and engraftment samples from the same subjects showed that the HSCT event resulted in a decrease in the relative abundance of several genera important for a symbiotic, health-promoting relationship between GM and host, namely *Faecalibacterium, [Ruminococcus], Roseburia, Lachnospira, Dorea, Coprococcus, Blautia* (paired Wilcoxon test, *P* < 0.05) (Fig. 3c, d, e). In relation to the subsequent development of aGvHD, we found that in subjects developing gut grade II-IV aGvHD (Fig. 3e) the changes occurring after HSCT were less evident than in all other subjects (Fig. 3c and d) and not significant, hinting that those well-known health-promoting bacterial groups were already less relatively abundant on average before HSCT, even if the comparison with the other pre-HSCT samples showed significant differences only for *Blautia* (Fig. 2c, Additional file 2: Table S1). This might be related to the higher pre-HSCT variability found in subjects who developed gut aGvHD (Additional file 1: Figure S6), which might have hindered the detection of common dysbiotic features.

### Analysis of microbiota variability > 30 days after HSCT

PCoA analysis based on genus-level relative abundances showed no separation between samples taken > 30 days after HSCT from subjects who subsequently either did or did not develop aGvHD (adonis test, *P* > 0.05) (Additional file 1: Figure S7). Random Forest analysis confirmed the absence of significant difference in the post-HSCT GM profile among patients developing either skin or gut aGvHD (error rate, 55%).

## Discussion

The study presented here is a multicenter, longitudinal observation of microbiota dynamics in pediatric patients undergoing HSCT for a variety of hematological diseases. The pediatric cohort also included children aged 2 years or less, whose intestinal ecosystem is known to be highly dynamic in its progressive “maturation” toward the typically adult GM structure [30, 31]. As a confirmation of the peculiarity of this phase of life for the GM assembly, in our study, samples from 2-year-old or younger patients were found to significantly separate from all other samples, both before HSCT and 1 month after the procedure. Interestingly, this was not true for the samples taken at engraftment, highlighting the extensiveness of the perturbation induced on the GM structure by HSCT that temporarily cancels the age-dependent signature in the intestinal microbial composition.

When we explored the impact of different hospitals in which patients were treated, the relative abundance of few genera was found discriminating for the enrollment center, and this was taken into account in interpreting the results of the subsequent analysis and evaluating their significance. The detected discriminant genera (e.g. *Enterococcus*, *Citrobacter*, and other members of Enterobacteriaceae, such as *Enterobacter* and *Klebsiella*) are opportunistic members of the human GM, whose presence could possibly be related to the hospital environment itself. Indeed, *Citrobacter*, *Enterococcus, Klebsiella* and *Enterobacter* are well-known possible sources of nosocomial bloodstream infections that are an important cause of morbidity and mortality in children undergoing HSCT [26–29, 32]. Patient’s colonization and hospital dissemination of potentially multidrug-resistant bacteria are considered a worrisome phenomenon in hematological patients [33]. Considering that Enterobacteriaceae are known for their potential for rapid dissemination in healthcare settings and because they can cause severe infections, inter-hospital GM differences should be checked for in all future multi-center studies and taken into account in routine clinical practice and patient management [29]. Nevertheless, the relative abundance of these genera was not found significantly associated with the subsequent development of aGvHD, so these center-related variations should not have biased the results of our study.

Despite the complexity of this study in terms of possible confounding variables (i.e. chemotherapy, antibiotics, proton-pump inhibitors, and hospitalization), it was possible to detect a signature of the future development of aGvHD in the GM composition before HSCT. More precisely, lower relative abundance of *Blautia* was associated with the subsequent aGvHD development, confirming, in pediatric patients, previous observations obtained in adults associating this symbiotic, acetate-producing genus with lower occurrence of aGvHD [8, 34]. Curiously, a recent work based on a large number of Japanese patients found no differences in *Blautia* abundance between aGvHD and non-aGvHD subjects [35]. However, as stated by the authors themselves, the influence of lifestyle and nutrition on the GM composition might play a role in determining the differences in GM response among populations. Indeed, the importance of relying on localized baselines in studies focused on GM and disease has recently been stressed, [36] and *Blautia* has been identified as one of the taxa most influenced by the degree of urbanization [37].

On the contrary, higher pre-HSCT relative abundances of *Fusobacterium nucleatum* were detected in the subgroup of subjects who subsequently developed aGvHD with gastrointestinal involvement. *Fusobacterium* has never been linked to aGvHD occurrence in previous studies, but it has been reported to play an etiological role in intestinal inflammation and colon cancer, [38–40] also in children, as in the case of pediatric Crohn’s disease and appendicitis [41, 42]. The ability of *Fusobacterium* to trigger the secretion of TNF-alpha and IL-6 has been demonstrated by in vitro studies [43]. As stated by Zeiser et al. [2] and Chen et al. [44], these cytokines might recruit donor T cells responsible for aGvHD complications. It is also worth mentioning that the pre-HSCT GM of subjects who did not develop aGvHD tended to show higher biodiversity and, coherently, higher occurrence of several highly subdominant bacterial groups, among which members of the Barnesiellaceae family were reported. Interestingly, it was recently hypothesized that the presence of *Barnesiella* in the gut environment of HSCT patients might be protective against proliferation of vancomycin-resistant *Enterococcus*, one of the most important pathogens inducing HSCT-related morbidity/mortality [6, 32, 45, 46].

The peculiarities of the pre-HSCT GM configuration could, thus, contribute to the outcome of the immunological recovery following HSCT. In particular, the higher ecosystem diversity, an increased relative abundance of the symbiotic *Blautia* and lower relative abundance of the known pathobiont *F. nucleatum* may protect from severe aGvHD. These findings confirm the importance of a healthy-like GM profile, i.e. a diverse syntrophic ecosystem enriched in symbiotic species and deprived of potentially pro-inflammatory members, to face events that are microbiologically, immunologically and metabolically challenging for the host, such as HSCT and related procedures.

Interestingly, we found that the changes induced by HSCT were less significant in subjects who developed aGvHD with gastrointestinal involvement. An explanation of this might reside in the low number of subjects with gut aGvHD enrolled, but also in the greater inter-individual variability in the GM composition shown by these subjects with respect to non-aGvHD and skin-only aGvHD subjects. It might be tempting to hypothesize that the GM of subjects developing gut aGvHD could have been already dysbiotic before HSCT, with a strong individual variation. These observations confirm previous findings on inflammatory bowel disease patients, whose GM was shown to fluctuate much more than in healthy individuals, resulting in high inter-patient variation [47]. This could be related to what has been recently defined as the “Anna Karenina principle”, stating that each dysbiotic microbial ecosystem is dysbiotic in its own way [48]. Such a pattern could have hindered commonalities in HSCT-related variations.

At engraftment, patients with gut grade II-IV aGvHD showed increasing *Bacteroides* relative abundance, indicating a possible association between gut aGvHD and early GM changes following HSCT. However, the number of children developing gut grade II-IV aGvHD was low and unevenly distributed among hospitals: three at the hospital of Verona, with gut aGvHD diagnosed before engraftment sampling took place, and three at the hospital of Bologna, diagnosed after engraftment sampling. No gut aGvHD samples were available from Rome and Pavia hospitals. While this might have influenced the results presented here, it highlights a possible role that hospital-specific environmental bacteria might play in favoring the onset and/or progression of aGvHD complications. Indeed, OTUs assigned to the genus *Erwinia*, and re-assigned to the known nosocomial species *Enterobacter cloacae,* showed significantly higher levels in samples taken at the Verona hospital than all the other hospitals, and this feature was particularly evident in engraftment samples of patients developing gut aGvHD.

Finally, samples taken 30 days after HSCT did not show significant differences in the gut ecosystem structure between children who either did or did not develop aGvHD. We can, thus, suggest that early events in the GM-host symbiosis are the ones that mostly impact on donor-versus-host alloreactivity.

## Conclusions

In the model we propose, the composition and diversity of the GM, as it is before HSCT takes place, might lack features of a healthy-like community, such as a sufficient relative abundance of saccharolytic symbionts as *Blautia*, or include risk factors for localized mucosal damage, such as a higher presence of *Fusobacterium nucleatum*, thus losing its resilience. Such GM might have more chances, during pre-HSCT hospitalization and the HSCT procedure, to evolve into a “pathobiome”, i.e. a “health-care adapted pathogenic community”, [49] that is unable to support immunological recovery in pediatric patients. Even if the molecular mechanisms involved in this process still need to be explored in further studies by increasing the total number of cases, our data may help support a rational approach to fecal material transplantation in HSCT. Indeed, the association between GM configuration and gut aGvHD onset may help to stratify pediatric patients according to the risk of developing gut aGvHD and, eventually, to rationally select the best stool donor for a fecal material transplantation approach able to provide the patient a beneficial, aGvHD-protecting microbiota.

## Additional files

Additional file 1: **Figure S1.** PCoA based on Bray-Curtis distances of genus-level relative abundance profiles of the samples sequenced in the present study using Illumina HiSeq and those from the study of Biagi et al. [7] sequenced by Roche 454 pyrosequencing. **Figure S2.** PCoA based on Bray-Curtis distances of genus-level relative abundance profiles of samples taken from patients aged < 2 years, 2–11 years and > 12 years, before, 12–28 days after, and > 30 days after HSCT. **Figure S3.** PCoA based on Bray-Curtis distances of genus-level relative abundance profiles of samples taken from patients hospitalized in the four centers before, 12–28 days after, and > 30 days after HSCT. **Figure S4.** Box and whiskers distribution of the relative abundance (%) of OTUs assigned to the genera *Enterococcus*, *Citrobacter* and *Erwinia*, in samples grouped as pre-HSCT, engraftment and post-HSCT, for the four enrollment centers. **Figure S5.** Box and whiskers distribution of the Simpson diversity index calculated for the genus-level microbiota profiles in samples taken before HSCT from subjects who did not develop aGvHD, who developed aGvHD (I-II grade) at a skin level, and who developed gastrointestinal aGvHD (II-IV grade). **Figure S6.** Box and whiskers distribution of the Bray-Curtis distances calculated using the genus-level gut microbiota profiles of samples taken before HSCT from subjects who did not develop aGvHD, who developed aGvHD (I-II grade) at a skin level, and who developed gastrointestinal aGvHD (II-IV grade). **Figure S7.** PCoA based on Bray-Curtis distances of genus-level relative abundance profiles of samples taken > 30 days after HSCT from patients who did not develop aGvHD, who developed aGvHD (I-II grade) at a skin level, and who developed gastrointestinal aGvHD (II-IV grade). (PDF 305 kb)

Additional file 2: **Tables S1.** Average relative abundances in pre-HSCT and engraftment samples, of the main discriminant genera between the two time points in subjects who did not develop aGvHD (non-aGvHD), who developed aGvHD (I-II grade) at a skin level (Skin aGvHD), and who developed gastrointestinal aGvHD (II-IV grade) (Gut aGvHD) (Wilcoxon test, *P* ≤ 0.05, in bold, in at least one group of subjects). *P* values between 0.05 and 0.1 are reported in italics, whereas P values > 0.1 are reported as “ns”, not significant. (PDF 257 kb)

## Abbreviations

aGvHD: acute Graft-versus-Host Disease
GM: Gut Microbiota
HSCT: Hematopoietic Stem Cell Transplantation
