# Supplementary material for: Early gut microbiota signature of aGvHD in children given allogeneic hematopoietic cell transplantation for hematological disorders
Source: BMC Med Genomics. 2019 Mar 7;12:49. doi: 10.1186/s12920-019-0494-7 (PMC6404274; doi:10.1186/s12920-019-0494-7)

Additional figures

Figure S1. PCoA based on Bray-Curtis distances of genus-level abundance profiles of the samples sequenced in the present study using Illumina HiSeq (red) and those from the study of Biagi *et al.* (2015) sequenced by Roche 454 pyrosequencing (yellow). Samples are identified by filled circles. PCoA first and second principal components are plotted.

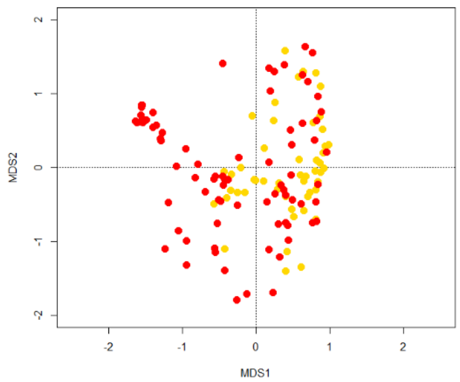

Figure S2. PCoA based on Bray-Curtis distances of genus-level abundance profiles of samples taken from patients aged <2 years (pink), 2-11 years (yellow) and >12 years (green), before HSCT (pre, A), 12-28 days after HSCT (engraftment, B) and >30 days after HSCT (post, C). Samples are identified by filled circles. PCoA first and second principal components are plotted.

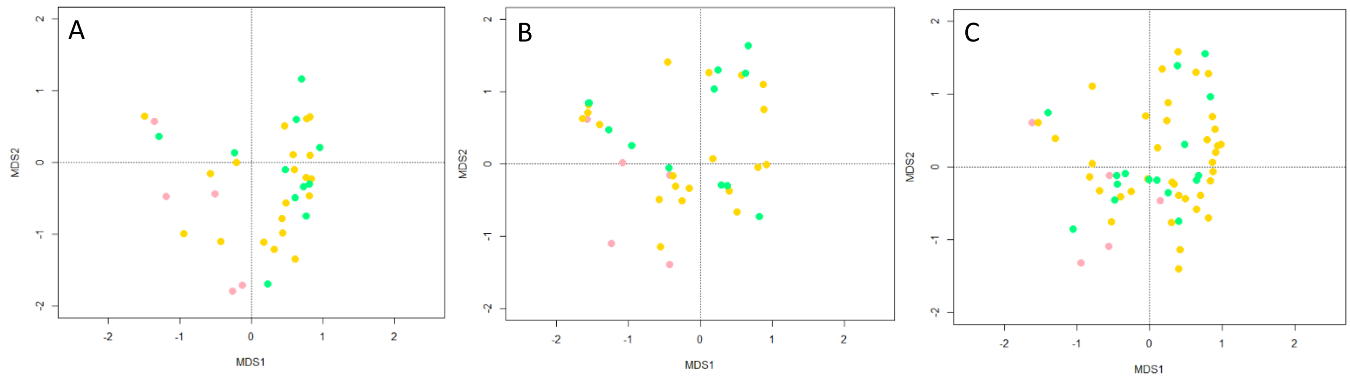

Figure S3. PCoA based on Bray-Curtis distances of genus-level abundance profiles of samples taken from patients hospitalized in the center of Bologna (light blue), Pavia (hotpink), Rome (gold), and Verona (green), before HSCT (pre, A), 12-28 days after HSCT (engraftment, B) and >30 days after HSCT (post, C). Samples are identified by filled circles. PCoA first and second principal components are plotted.

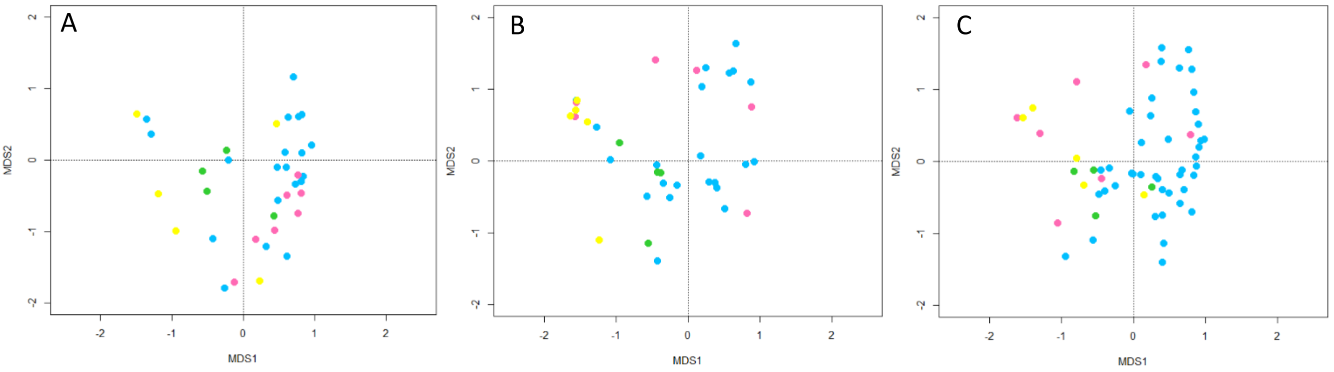

Figure S4. Box and whiskers distribution of the relative abundance (%) of OTUs assigned to the genera *Enterococcus* **(A, D, G, L)**, *Citrobacter* **(B, E, H, M)** and *Erwinia* **(C, F, I, M)**, in samples grouped as pre-transplantation (PRE, green), engraftment (ENG, orange) and post-transplantation (POST, blue), for the four enrollment centers of Bologna **(A, B, C)**, Pavia **(D, E, F)**, Verona **(G, H, I)** and Rome **(L, M, N)** hospitals.

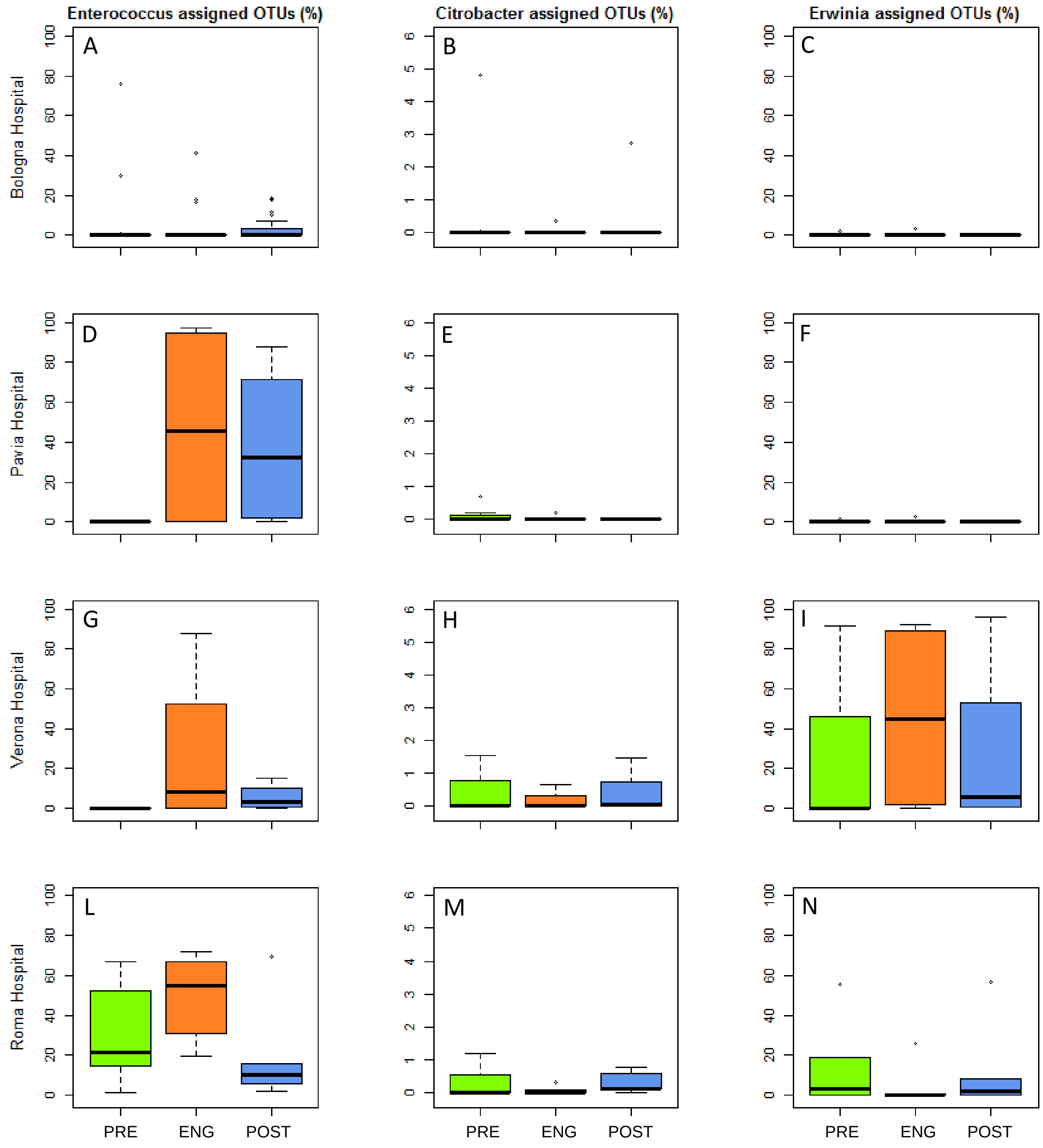

Figure S5. Box and whiskers distribution of the Simpson diversity index calculated for the genus-level microbiota profiles in samples taken before HSCT from subjects who did not develop aGvHD (green), who developed aGvHD (I-II grade) at a skin level (gold), and who developed gastrointestinal aGvHD (II-IV grade) (dark red).

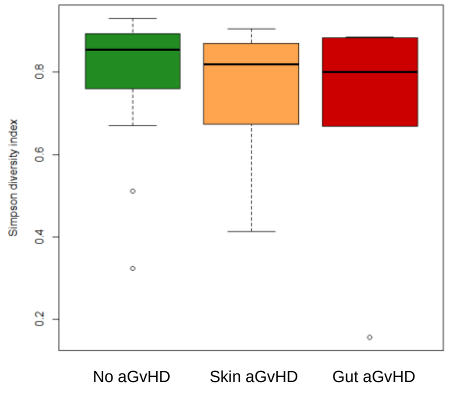

Figure S6. Box and whiskers distribution of the Bray-Curtis distances calculated using the genus-level gut microbiota profiles of samples taken before HSCT from subjects who did not develop aGvHD (green), who developed aGvHD (I-II grade) at a skin level (gold), and who developed gastrointestinal aGvHD (II-IV grade) (dark red).

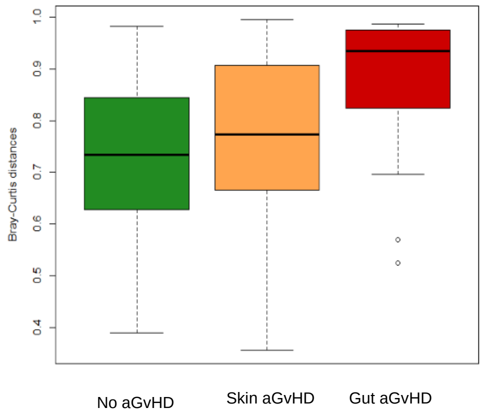

Figure S7. PCoA based on Bray-Curtis distances of genus-level abundance profiles of samples taken >30 days after HSCT from patients who did not develop aGvHD (green), who developed aGvHD (I-II grade) at a skin level (gold), and who developed gastrointestinal aGvHD (II-IV grade) (dark red). Samples are identified by filled circles. First and second principal components (MDS1 and MDS2) are plotted, accounting for 18.4 and 8.9% of variance in the dataset, respectively.

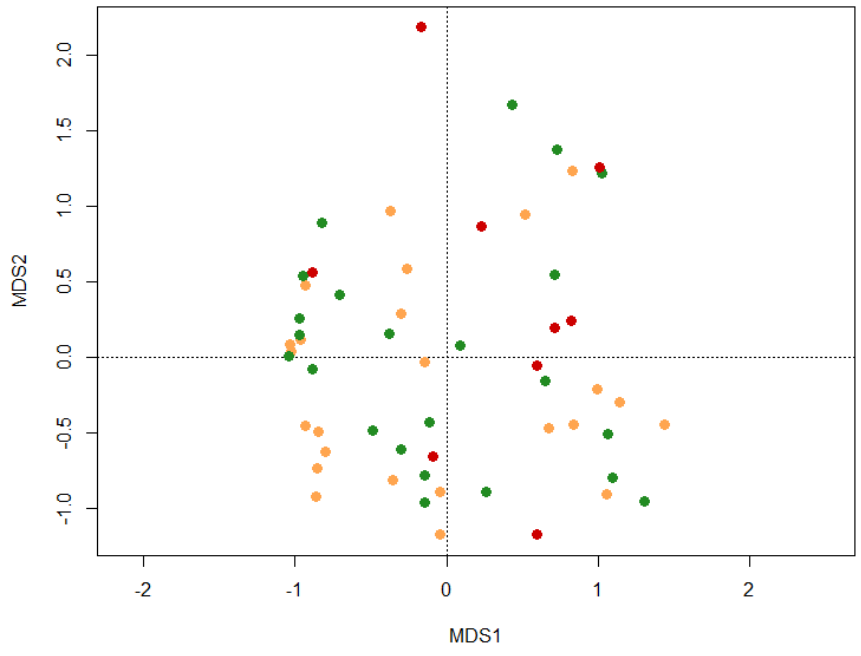

Supplement: Supplementary file 1 — Figure S1. PCoA based on Bray-Curtis distances of genus-level relative abundance profiles of the samples sequenced in the present study using Illumina HiSeq and those from the study of Biagi et al. [7] sequenced by Roche 454 pyrosequencing. Figure S2. PCoA based on Bray-Curtis distances of genus-level relative abundance profiles of samples taken from patients aged < 2 years, 2–11 years and > 12 years, before, 12–28 days after, and > 30 days after HSCT. Figure S3. PCoA based on Bray-Curtis distances of genus-level relative abundance profiles of samples taken from patients hospitalized in the four centers before, 12–28 days after, and > 30 days after HSCT. Figure S4. Box and whiskers distribution of the relative abundance (%) of OTUs assigned to the genera Enterococcus, Citrobacter and Erwinia, in samples grouped as pre-HSCT, engraftment and post-HSCT, for the four enrollment centers. Figure S5. Box and whiskers distribution of the Simpson diversity index calculated for the genus-level microbiota profiles in samples taken before HSCT from subjects who did not develop aGvHD, who developed aGvHD (I-II grade) at a skin level, and who developed gastrointestinal aGvHD (II-IV grade). Figure S6. Box and whiskers distribution of the Bray-Curtis distances calculated using the genus-level gut microbiota profiles of samples taken before HSCT from subjects who did not develop aGvHD, who developed aGvHD (I-II grade) at a skin level, and who developed gastrointestinal aGvHD (II-IV grade). Figure S7. PCoA based on Bray-Curtis distances of genus-level relative abundance profiles of samples taken > 30 days after HSCT from patients who did not develop aGvHD, who developed aGvHD (I-II grade) at a skin level, and who developed gastrointestinal aGvHD (II-IV grade). (PDF 305 kb) [file 12920_2019_494_MOESM1_ESM.pdf]
