# Supplementary material for: Early gut microbiota signature of aGvHD in children given allogeneic hematopoietic cell transplantation for hematological disorders
Source: BMC Med Genomics. 2019 Mar 7;12:49. doi: 10.1186/s12920-019-0494-7 (PMC6404274; doi:10.1186/s12920-019-0494-7)
Supplement: Supplementary file 2 — Tables S1. Average relative abundances in pre-HSCT and engraftment samples, of the main discriminant genera between the two time points in subjects who did not develop aGvHD (non-aGvHD), who developed aGvHD (I-II grade) at a skin level (Skin aGvHD), and who developed gastrointestinal aGvHD (II-IV grade) (Gut aGvHD) (Wilcoxon test, P ≤ 0.05, in bold, in at least one group of subjects). P values between 0.05 and 0.1 are reported in italics, whereas P values > 0.1 are reported as “ns”, not significant. (PDF 257 kb) [file 12920_2019_494_MOESM2_ESM.pdf]

Table S1. Average relative abundances in pre-HSCT and engrafment samples, of the main discriminant genera between the two time points in subjects who did not develop aGvHD (non-aGvHD), who developed aGvHD (I-II grade) at a skin level (Skin aGvHD), and who developed gastrointestinal aGvHD (II-IV grade) (Gut aGvHD) (FDR corrected paired Wilcoxon rank-sum tests P ≤ 0.05, in bold, in at least one group of subjects). P values between 0.05 and 0.1 are reported in italic, whereas P values >0.1 are reported as "ns", not significant.

	Pre-HSCT samples (average rel. ab. %)			Engrafment samples (average rel. ab. %)			Paired Wilcoxon test (P value)		
	Non-aGvHD	Skin aGvHD	Gut aGvHD	Non-aGvHD	Skin aGvHD	Gut aGvHD	Non-aGvHD	Skin aGvHD	Gut aGvHD
Bifidobacterium	11.4	13.4	7.2	6.5	0.7	8.1	ns	**0.033**	ns
Blautia	11.8	5.3	7.5	2.0	0.7	1.1	**0.00013**	ns	ns
Coprococcus	2.5	1.6	0.3	0.7	0.5	0.2	*0.086*	**0.045**	ns
Dorea	1.0	1.1	2.1	0.3	0.6	0.3	**0.025**	**0.013**	ns
Lachnospira	0.6	0.2	0.6	0.02	0.01	0.09	**0.0039**	**0.028**	ns
Roseburia	4.3	5.4	2.9	0.9	0.8	0.7	**0.034**	**0.036**	ns
[Ruminococcus]	5.7	8.3	1.5	2.6	5.4	1.3	*0.087*	**0.043**	ns
Faecalibacterium	6.6	8.1	5.7	1.6	0.3	0.3	**0.0029**	ns	*0.071*
Oscillospira	0.9	1.6	1.6	0.5	0.2	1.3	**0.027**	**0.011**	ns
